# The Application of an Augmented Gravity Model to Measure the Effects of a Regionalization of Potential Risk Distribution of the US Cull Sow Market

**DOI:** 10.3390/vetsci9050215

**Published:** 2022-04-28

**Authors:** Benjamin Blair, James Lowe

**Affiliations:** 1 Department of Pathobiology, University of Illinois College of Veterinary Medicine, Urbana, IL 61802, USA; bblair2@illinois.edu; 2 Lowe Consulting, Champaign, IL 61853, USA

**Keywords:** risk, swine, disease, cull, movements, trade, sow, gravity model

## Abstract

The continuous threat of foreign animal disease (FAD) is real and present for the U.S. swine industry. Because of this, the industry has developed plans to ensure business continuity during a FAD outbreak. A core aspect of these plans is regional standstill orders of swine movements to prevent disease spread following a FAD introduction. Unfortunately, there is a dearth of information about the impact of such practices on animal movements throughout the remaining swine marketing channel. This study utilizes a simplified gravity model, to understand the effects of standstill orders on individual states. The effect of each closure on the established trade patterns is determined by monitoring changes in a PPML regression coefficients of the model. Model validation compared the predicted impact of the closure of a terminal processing facility against a real-life closure dataset collected during the SARS-CoV-2 pandemic. The analysis determined that both the population size and location of the closure affected the observed trade patterns. These findings suggest that using a regional stop movement order may complicate disease introduction preparation as each policy comes with its own potential outcome, shifting the geospatial distribution of area risk posed by these cull populations.

## 1. Introduction

The size of the US swine industry, with more than six million breeding animals [1], in conjunction with its diverse geographical distribution requires a complex set of movements to maintain the sustainability and continuity of the marketing channels [2]. The movement of cull sows (breeding females who are destined for slaughter) through the marketing channel and into the human food chain is exceptionally complex [3]. Within the cull sow marketing channel, animals move from farms across the US and Canada to a limited number (18) of cull sow-specific slaughter facilities which are not geographically distributed proportionally to the location of the swine breeding herds. This requires a system where the mixing and movement of sows across the country is common [3]. The cull sow system is different from the lean hog market [4], the primary pork supply chain, where hogs move directly to terminal processing facilities, cull sows move through at least one or more additional points before slaughter [2].

The cull sow marketing channel is composed of three unique components: farms of origin, collection points, and terminal processing facilities [4]. The marketing channel’s unique segment, intermediary collection points, exists for one reason, to generate additional value for the industry. Collection points continuously buy animals from farms, then sort sows by weight and body type to sell them to the proper slaughter facility based on preference [4]. By allowing sow farms to remove small batches of cull animals at convenient times, collection points relieve the farm of the additional expense associated with retaining non-productive sows [5,6].

Traditionally, the cull sow marketing channel was assumed to be transient, composed of a population of animals moving quickly from farm of origin to terminal processing facility. These assumed attributes were thought to have only a minor material impact on the amplification or transmission of disease within the US swine industry. However, recent disease outbreaks within the industry, such as PEDv [7] and Seneca Valley virus [8], have brought forward new questions regarding this marketing segment’s role in disease transmission and dissemination [9]. During these outbreaks, spread within marketing populations and outward to the industry was observed [9], raising concerns regarding this marketing network’s role in disease transmission.

Recent studies have hypothesized that this population is not transient, but instead a dynamic population capable of incubating and disseminating pathogens [3]. This hypothesis is predicated partially on the extended time sows remain within the marketing channel [3]. While this time varies, the median time spent within the marking channel is three days [3], with more than 8.9% of sows remain within the market for more than five days prior to slaughter [3]. Longer than the incubation period of many common swine diseases [10,11,12], these extended interactions within the marketing population allows for pathogen amplification and transmission, allowing this population to serve as a disease reservoir for the entire industry. In addition to the extended transit times, the complexity of movements occurring within the marketing channel influences this population’s inherent risk to the industry [3]. The complexity of these movements generates an increase in the number of connections within the marketing channel and thus indirection connections between farms of vastly different regions [3]. The act of selling into a marketing channel alone may increase the number of indirect connections to a farm by more than 50% [13]. Because these cull populations, associated with the terminal processing facilities, possess attributes common amongst all herds, a group of animals that interact for a period of time, they should be considered their own, independent herd. As such, these herds present risk to the industry just as any other swine herd would.

These herds, composed of cull animals, possess an associated disease status that impacts neighboring populations and the geographical region as the facility interacts with the industry. Populations, such as sow farms, are shown to significantly impact the disease risk of other swine populations within the same neighborhood or geographic vicinity [14]. The disease status of a sow farm’s animal population was one of the significant predictors of disease outbreaks in neighboring farms [14]. Suppose this is true of all populations that interact with a region or population. In that case, this herd of cull sows likely influences the area disease risk both regional and nationally, depending on its interactions with the swine industry. These neighboring effects have been observed between herds with varying disease outbreaks, Classical Swine Fever, PEDV, and PRRSV. Because of this, understanding the interactions these terminal processing facilities associated herds have with the industry is of utmost importance when trying to mitigate the effects of these poorly managed cull populations.

While it can be assumed these herds, associated with terminal processing facilities, present area disease risk, the amount of risk posed by each is largely unknown and difficult to calculate. However, the geographical distribution of this risk, as defined by their interactions, is known and has been shown to remains relatively stagnant under normal market conditions [3]. The consistency of this risk’s distribution makes mitigation possible as disease traceback and targeted disease surveillance can be enacted. However, while currently stagnant, the impact of a disruption to the industry, such as foreign animal disease, policy change, or market disruptions, to these geospatial distributions remain largely unknown. Changes to this distribution of risk threaten the swine industry, as the complexity and the changing dynamic of the market channel would allow disease to move erratically through the US. These previously undescribed dynamics complicate preparations for a foreign animal disease introduction. Changing interactions may allow pathogens to spread across the industry in ways previously unobserved, making mitigation attempts impossible.

With the increasing threat of foreign animal disease introduction to the US swine industry, understanding these potential shifts of the market populations’ geospatial risk distribution is of the utmost importance. This information may inform decisions regarding disease mitigation and control, ensuring the best outcome for the industry as a whole. As mentioned, the US swine industry has spent considerable effort safeguarding the country from ASF and preparing for its introduction. Across the globe, pork production has been devastated by African Swine Fever (ASF) and Foot and Mouth Disease (FMD) [15,16,17]. The recent global outbreak of ASF was first identified in Georgia in 2009, spreading across Europe and eventually China by August 2018 [18]. To combat this, the USDA released the “Disease Response Strategy African Swine Fever” in the spring of 2019 [19] to serve as strategic guidance in the event of an ASF outbreak. One of the critical pillars of response is the control and subsequent eradication of the disease. The goals of this process are three-fold:

(1) Detect, control, and contain the disease in animals as quickly as possible;
(2) Eradicate the disease using strategies that seek to stabilize animal agriculture, the food supply, and the economy and to protect public health and the environment;
(3) Provide science- and risk-based approaches and systems to facilitate continuity of business for non-infected animals.

As stated in the report, “*Quarantine and movement control measures are fundamental to any ASF response effort*” [19]. While a nationwide standstill on animal movements will ensure no disease transmission results, the effects of regionalized standstill orders are poorly understood. This is especially true concerning how a regionalized standstill will change the geospatial distribution of disease risk area posed by herds present within the cull sow marketing network. This study uses a simple gravitational model, a method commonly used within trade, to study the dynamic effects of regionalized stop movement practices. It will investigate how both known and unknown market influences on sow movements impact the distribution of area disease risk posed by these marketing populations.

Because the interactions within the cull sow marketing channel can be viewed as a form of trade, the use of the augmented gravity model makes sense when exploring system dynamics in the face of disruptions. Economists have long quantified dynamic trade patterns under the influence of behavior or policy through gravitational models [20,21,22,23]. Walter Isard first introduced the use of the augmented gravity theory in 1954 [24] as an empirical method to investigate the flow of commodities from different distances. While many doubted the theoretical justification for augmented gravity theory, its trade application continues to serve as an accurate method to understand the movement of goods.

As a standard method to study bilateral trade flows, researchers have implemented augmented gravity modeling for decades within international economics [21,25,26,27,28]. Traditionally studies have employed this method of econometric analysis to understand trade patterns. Within these models, weight is assigned based on the size of each parties’ economy, while distances represent trade cost, commonly expressed as a combination of physical distance and obstacles to trade. The gravitational theory of trade assumes that the closer and more massive trade partners’ economies are, the more trade between them [24].

Many studies exist utilizing augmented gravity modeling to evaluate the effects of trade between two entities. One such study investigated the South Asian Free Trade Area (SAFTA) role and how it potentially impacts countries’ exports [29]. It found that while some countries, Pakistan and India, were expected to gain exports from joining the agreement, others, such as Sri Lanka, were not [29]. Another study empirically quantified the trade potential between India and 146 countries [30]. It found that the economy’s size and cultural similarities impact bilateral trade [30]. It also proposed changes to policies allowing trade to double with China.

The application of the theory also includes animal movements. One such study focuses on the impact of EU-mandated country of origin labeling on Italy’s cattle movement [23]. Through use of a gravity model it found that regions of the most extensive inventory are more likely to trade with each other, regardless of distance than those with small populations [23]. Researchers have also used augmented gravity modeling to assess the impact of disease outbreaks in the US on poultry and poultry product trade [31]. Both studies show that the gravity model’s use could quantify various impacts on an industry, such as disease, on bilateral trade.

While augmented gravity models have been used exhaustively for international and local trade agreements, their application to understanding animal marketing is limited and within swine nonexistent. While some work exists [23,31], none have focused on movement within the US swine industry. This study implements the augmented gravity model to study the effects of regionalized standstills on animal movement in the cull sow marketing channel. Thus, the dynamic impact of policy changes on the geographical distribution of disease dissemination risk posed by the marketing channel.

## 2. Materials and Methods

### 2.1. Data Collection

Data collection was in partnership with the USDA-APHIS-VS Federal Brucellosis Laboratory (Laboratory). This laboratory collected all premises identification number tags (PIN) [32] affiliated with samples submitted for surveillance of brucellosis, representing the sows randomly sampled from seven US terminal processing facilities as part of the national program for brucellosis and pseudorabies surveillance.

PIN currently serves as the traceability method of the “Swine ID Plan” established by the industry in 2004. The industry has a high PIN tag adoption rate, with PIN tags present in greater than 90% of sows at the time of slaughter [2]. Samples collected by APHIS originate from seven US processing facilities. This study refers to the terminal processing facilities as F1-F7 to protect the identity of the participating facilities.

PIN collection occurred over six weeks with collection one week per month in May, June, July of 2018, and February, March, April of 2019, with the recording of the management/sow ID, Premise ID, state, plant, and kill date from each PIN. Geolocations for Premise ID came from a pork checkoff premise lookup database allowing for the acquisition of the street address for each premise ID. Google Maps [33] was then used to confirm a swine facility at the corresponding street address and to convert the address into latitude and longitude coordinates.

From the 16 states with the largest sow populations, an annual cull population was estimated at 50% of the sow inventory, as reported by the National Agriculture Statistics Service in the national hogs and pigs report [1]. US pork checkoff reports provided capacity estimates for five of the seven terminal processing facilities [34]. The capacities of two plants, F6 and F7, are unknown, resulting in their exclusion from the dataset. The geolocation for each state was denoted by the state’s capitol, while the terminal processing facilities’ geolocation reflects the business’s physical location.

### 2.2. Mathematical Description of the Cull Marketing Network

The relationships that exist within the cull sow marketing network are complex and challenging to understand. Terminal processing facility populations attract sows from many states at varying rates. Describing this phenomenon through the use of analogies helps us make sense of such complex relationships. The use of gravitational theory is one such analogy. This analogy has long been used within trade and policy discussions to understand bilateral trade relationships between different countries:

F = G (M_1_ × M_2_)/d

The above equation has been used to describe the force of attraction (gravity) one body has on another. It describes the complicated relationship, Force (F), that objects exert on each other as a function of the product of their masses (M1 and M2); this is inversely proportional to the distance between them (d) and the constraints of the system or universe represented by the gravitational constant (G). This equation also works intuitively to describe trade networks, as trade (X_ij_) is dependent on the size of economies (Y_i_, Y_j_) and distance or trade cost between trading partners (t_ij_), with C serving as a constant, as described in this simplified equation:

X_ij_ = C (Y_i_ Y_j_)/t_ij_

The equation was modified to improve the empirical application of this equation. Modifications allow variables to impact trade at varying degrees:

X_ij_ = C (Y_i_^b1^ Y_j_^b2^)/(t_ij_^b3^)

This relationship is commonly represented empirically as:

ln(X_ij_) = C + b_1_ ln(Y_i_)+b_2_ ln(Y_j_) + b_3_ ln(t_ij_) + e_ij_….

This study uses this empirical equation combined with the data collected to understand the complicated relationship between a state’s sow population and terminal processing facilities. For this study’s simple gravity model, X_ij_ is equal to the total trade between a state and terminal processing facilities. Y_i_ and Y_j_ represent the economy’s size or the weekly cull sow population or slaughter capacity for the states and terminal processing facilities, respectively. This model’s trade cost (t_ij_) represents the Euclidian distance between the state’s capitol and terminal processing facilities and the reported price difference, between regions reported by the Daily Direct Prior Day Sow and Boar report [35]:

ln(Trade_ij_) = C+b_1_ ln(Pop_i_) + b_2_ ln(Pop_j_) + b_3_ ln(Distance_ij_) + b_4_ ln(Basis_ij_)+e_ij_….

Due to the multiple zero trade values, this study employs Poisson regression by pseudo maximum likelihood (PPML) to fit the linear model for b_1_, b_2_, b_3_, and b_4_ through the use of the gravity package [36] within R [37].

Once the initial gravity model was complete, the potential trade index between each state and terminal processing facility was calculated for each week, as previously described in [22]. The potential trade index is simply the proportion of actual to predicted trade. This is done by taking the observed trade frequency over the fitted trade frequency of the PPML model:

$$\mathrm{Potential}\;\mathrm{Trade}\;\mathrm{Index}=\frac{{\mathrm{Trade}}_{\mathrm{ij}}}{{\mathrm{Trade}}_{îj}}$$

Trade_ij_ represents the observed trade between state and processing facility and Trade_îj_ represents the fitted trade frequency.

Trade potential, or the frequency of trades that can still be made to maximize current trading conditions can then be calculated by multiplying the observed trade frequency by one minus the trade index. Trade potential acts as a metric of potential trade creation between entities [21].

$$\mathrm{Trade}\;\mathrm{Potential}={\;\mathrm{Trade}}_{\mathrm{ij}}\times \left(1-\mathrm{Potential}\;\mathrm{Trade}\;\mathrm{Index}\right)$$

Trade Potential and Potential Trade Index values were calculated for each state-processing facility interaction.

### 2.3. Model Validation

Because this type of methodology has not previously been performed on swine trade networks, validation is key to further valid results. Disruptions within the cull sow marketing network resulted from the SARS-CoV-2 pandemic, allowing the opportunity to validate a model creation method by comparing observed data to theoretical simulation data after a terminal processing facility’s closure (F1). Disruption trade observations were collected in the same manner as previously described, occurring during the first three weeks of May 2020.

To create the simulation of a single processing facility’s closure, three weeks of data from the previous dataset were adjusted so that the trade to F1 was equal to zero. Then, by utilizing the previously calculated trade potential indices, reductions in demand were made. Reductions in trade were made equally amongst all relationships with a negative trade potential to account for an increase in supply or decrease in processing facility space. A PPML model, as previously described, then utilized both the observed disruption data and simulated data to generate the coefficient and standard error of each significant variable for comparison. Comparisons of the two models were made using Chow’s test to determine if the models were significantly different statistically. Statistical difference was concluded if the *p*-value was less than 0.05.

### 2.4. Stop Movement Scenarios

Using the mathematical relationship previously described, the PPML model was subject to two stop-movement scenarios; a stop-movement of North Carolina and Missouri populations. For each scenario, the trade from the standstill population to processing facilities was set to zero. Then, utilizing the potential trade index, adjustments were made to trade between remaining populations and terminal processing facilities by equaling the increase in demand between available potential trade. The study then uses the PPML of each unique scenario to quantify the value of each significant coefficient. The North Carolina swine population’s closure allows for the effects of a large population a great distance away from the centroid of the industry to be compared to a moderate-sized population near the centroid of the industry.

To better understand the impact of these model disruptions on the industry, heat maps of the 16 reported states were generated. A map showing the proportion of each state’s population entering the terminal processing facility’s population was then calculated by dividing the fitted outcome of the PPML model and each state’s population. In addition, for both the Missouri and North Carolina scenarios, a heat map representing the percent change to these proportions was generated for each of the five terminal processing facilities. These graphs better allow the reader to understand the impact of shifting coefficients within the PPML.

## 3. Results

### 3.1. Mathematical Description of the Cull Sow Marketing Network

The baseline empirical gravity model formed from the trade data collected between 2018 and 2019 fits all variable coefficients, with distance and populations remaining significant (Table 1). For the cull sow market during normal operating conditions, the distance between the terminal processing facility and state population, terminal processing capacity, state population all impact the observed trade (Table 1). A 1% increase in the distance between trade partners results in a decrease of 1.15% in trade, where increases in terminal processing facilities and state populations result in increased trade (Table 1).

**Figure 1:**
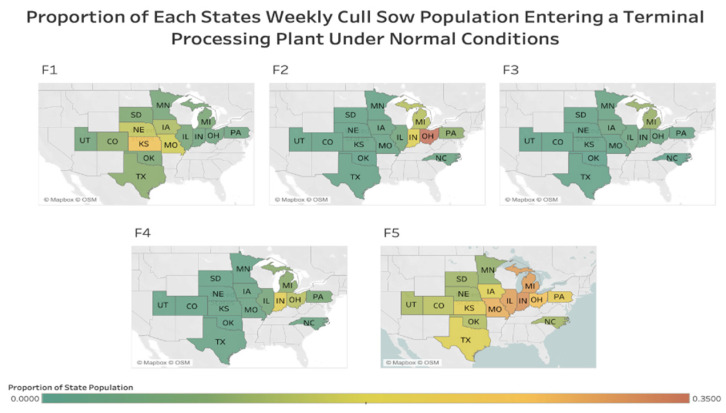
The proportion of a state’s cull sow population that enters each terminal processing facility F1–F5, under normal operating conditions. Each pane labeled F1, F2, F3, F4, F5 represents a different processing facility.

Under normal marketing conditions the maximum potential trade index observed was 1.12 between Colorado and F4. This suggest that the opportunity F4 has increased demand of sows from Colorado. While the minimum index observed was −1.59 between Nebraska and F3, representing a surplus of sows in Nebraska to enter the F3 facility. To better compare the supply/demand situation between states and facilities, the proportion of each state’s cull population entering the terminal processing facility population, as estimated by the fitted outcomes of the PPML, can be found in Figure 1.

### 3.2. Model Validation Results

The SARS-CoV-2 pandemic allowed for a unique insight into market dynamics in the wake of disruption. F1, a terminal processing facility within Kansas, closed for three weeks in May 2020. The closure of this terminal processing facility resulted in a mean increase of 4.66% of trade pattern variation. In comparison, the associated simulation based on the previous data resulted in a mean decrease in trade patterns of 1.02%.

### 3.3. Processing Facility Closure Scenario

The removal of 5500 sows weekly or the estimated cull sow capacity of F1, a terminal processing facility, allows for the validation of the model by comparing the real-life data set to the described disruption scenario creation method. The results of both models are present in Table 2.

The closure of the F1 processing facility decreased the impact of distance between the state population to terminal processing facilities. This impact increased by 33.7% and 22.4% for the simulation and actual data, respectively. A small impact was also observed within the terminal processing facility population’s impact on trade, as both the real scenario and simulation decreased by 5.06% and 9.78%, respectively. Finally, the impact of the state’s cull sow population on trade has decreased by 58.48% for the created simulation. The state population of cull sows falls out of the actual scenario’s completed model as it is no longer significant.

A comparison of both models reveals that the generalized change in coefficients is similar. While the fitted coefficients differ slightly from each other, the direction of change is the same within all coefficients. For the two coefficients that remain significant within both models, they lie within the standard error of each. Chow’s test also reveals that the regression models do not significantly differ with a *p*-value of 0.8396. This indicates that the gravity model of trade of the cull sow market is an adequate predictor of the impact of policy change or trade disruption.

### 3.4. North Carolina Scenario

**Figure 2:**
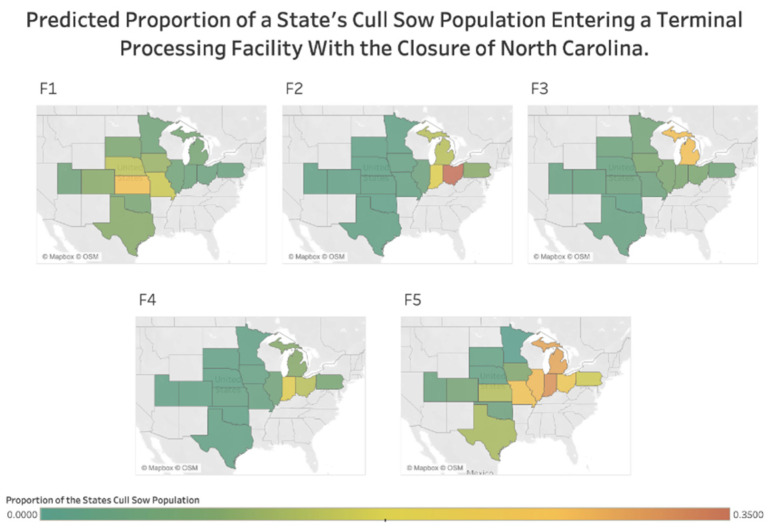
The predicted proportion of a state’s cull sow population that enters each terminal processing facility F1–F5, with a closure of movements to and from North Carolina. Each pane labeled F1, F2, F3, F4, F5 represents a different processing facility.

With the removal of 450,000 sows annually or the estimated cull sow population of North Carolina, changes to trade within the cull sow marketing network result in a mean decrease of terminal processing facilities purchasing variation of 12.69% with the simulated data. The simulated reduction in trade variation causes changes in the variable coefficients. A slight decrease in the coefficient associated with distance and facility capacity is present (Table 1). These changes result in an 11.16% decrease in the impact of distance on trade (Table 1). The effect of a processing facility capacity on the number of trades increases, while the state population’s impact decreased (Table 1) These results reveal that removing a large population a great distance from the center of the industry results in processing facilities purchasing animals close to their location, as seen with all terminal processing facilities (Figure 2).

Facilities with higher capacity also increase their activity as they try to buy sows to fill the standstill’s demand gaps, as seen especially with F5 (Figure 2). However, the state population’s size is less critical in determining potential trade partnerships in this scenario (Figure 2). Ultimately, during a disruption resulting in a loss of a major source of cull sows, large processing facilities must become more active in trying to fill the open spaces left by those removed animals. As a comparison between Figure 1 and Figure 2 shows, large terminal processing facilities, like F5, increase trading with states geographically near regardless of size and seek out fewer sows from states at greater distances.

### 3.5. Missouri Scenario

With the removal of 235,000 sows annually or the estimated cull sow population of Missouri, changes to trade within the cull sow marketing network result in a mean decrease in terminal processing facilities purchasing variation of 30.03% with the simulated data. With the simulated reduction in trade variation, massive changes in variable coefficients are present. An increase of the coefficient associated with distance and facility capacity is present (Table 1). These changes result in a 32.44% increase in the impact of distance on trade (Table 1). The effect of processing facilities’ capacity on the amount of trade decreased by 61.74%, while the state population’s impact is no longer significant within the model (Table 1).

**Figure 3:**
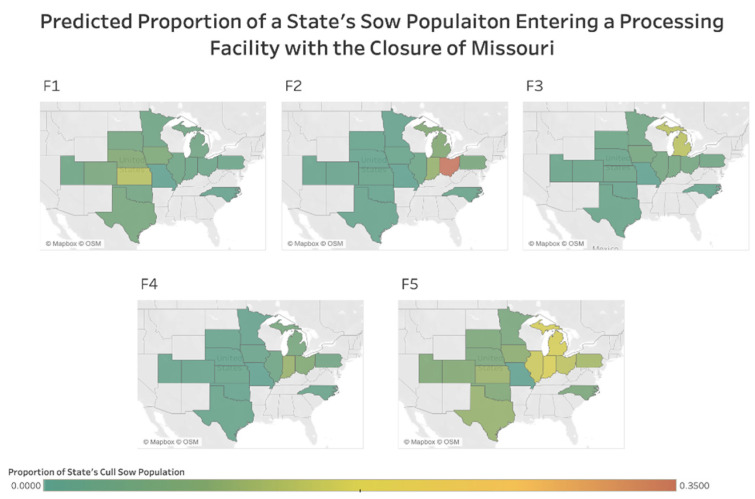
The predicted proportion of a state’s cull sow population that enters each terminal processing facility F1–F5, with a closure of movements to and from Missouri. Each pane labeled F1, F2, F3, F4, F5 represents a different processing facility.

These results reveal that removing a moderate-sized population within the center of the industry results in a substantial impact on trade such as removing a large population. The difference in scenarios show that location is just as influential on trade dynamics as the shutdown population’s size. As seen within Figure 3, the impact of distance from the terminal processing facility decreases as trade increases are observed in many states. Interestingly, especially compared to the North Carolina closure, removing the Missouri population causes the majority of the processing facilities to become more active in acquiring sows from many locations regardless of distance (Figure 3).

## 4. Discussion

To better describe the cull sow market’s network dynamics, a simple gravitational model was used to evaluate potential changes within the marketing system and how they may affect trade patterns between states and terminal processing facilities. The advantage of such a model is that it can provide empirical predictions of the system, as changes impact current system constraints. These impacts allow policymakers to evaluate how potential strategies, such as regional stop-movements or standstills, are related to disease spread implications regarding the geospatial distribution of area disease risk.

The SARS-CoV-2 pandemic has allowed the unique opportunity to validate using a gravitational model to predict the impact of a disruption to the cull sow marketing channel. During the pandemic, the closure of a terminal processing facility created the opportunity for the collection of a unique real-life disruption dataset. This unique dataset allows for the comparison of a simulated model to the real-life dataset’s results. The comparison results reveal that while some minor differences exist between the two models’ coefficients, overall, the models do not significantly differ. These results suggest that a gravitational model, simulated from previously collected data, serves as an effective means of understanding dynamic trade and distribution of interactions within this system.

Understanding both the cull sow marketing channel’s current dynamics and potential future dynamics is extremely important to quantify potential risk geographical distributions. Since most of the marketing risk is due to indirect contact within the channel and not directly from interactions between infected and susceptible animals, understanding the relative change in interactions between the terminal processing facilities’ cull populations and state populations is crucial during a pathogen spread event.

The current market’s trade dynamics are such that large populations within close proximity prefer to trade with each other. As shown, a 1% decrease in distance between entities results in a 1.15% increase in trade. However, as disruptions within the market occur, such as the potential closure of a region, these behaviors change. The scenario describing the closure of North Carolina allows for the impact that a large population lies a great distance from the industry’s centroid to be quantified. With a population of greater than 450,000 sows, the removal of this population results in a decrease in the influence of distance and state population size on trade, with a mild increase in terminal processing facility capacity. The removal of such a large population results in terminal processing facilities, especially larger facilities, preferring to buy from states closer to them regardless of state population size. Processing facilities trade at higher levels to meet demand, creating a more uniform distribution of trade between facilities and states, including states not evaluated in this project (Figure 2). Because this population lies so far from the center of the industry, no one terminal processing facility relies soley on this population to compose a significant proportion of their capacity. Thus, this population’s removal causes facilities to rely on previously well-defined local relationships to fill the gaps left by this closure. More extensive facilities such as F5 demand is slightly greater, due to size, and thus they most become more active across several states to fill that void.

The scenario describing the Missouri facility’s closure allows the opportunity to compare a population near the industry center. Because of Missouri’s proximity to the centroid of the marketing network, the simulation removing this population elucidates the location effect. The closure of Missouri results in a mean absolute change in coefficients of 45.81%. This scenario suggests an increase in the impact of distance. This means that terminal processing facilities are more likely to purchase sows from greater distances. The significant decrease observed in terminal processing facility population’s influence suggests that removing Missouri’s population would significantly increase demand in all terminal processing facilities regardless of capacity. This change in trade dynamics is so severe that the state population’s influence on trade is no longer significant. Unlike the closure of North Carolina, all processing facilities are impacted by removing a centrally located population near the major of the facilities, which causes meaningful changes in multiple facilities trade preferences, resonating throughout the entire system. This increase in demand at terminal processing facilities results in trade preferences with large state populations and nearby regions to dissolve. Demand in the 34 states, not reported, is likely to increase significantly as new relationships will be explored to remedy the increase in demand.

These scenarios show that an individual state’s closure can exacerbate the movement of pathogens as it may change the geospatial distribution of interactions and thus the distribution of risk to states. The changing geospatial distribution of interactions allows for unknown risk across the industry as the frequency of interactions between processing facility, and state populations become more uniform. This increasingly uniform distribution of interactions with states occurs as demand now outweighs supply, influencing facilities’ purchasing preferences, as defined by location, population size, and regional price difference that previously existed.

While the quantitative risk present from these interactions is unknown, changes in normal operating conditions cause the geographical distribution, which was relatively stagnant, to change depending on the location and size of the standstill population. These findings suggest that using a regional stop movement order may complicate disease introduction preparation. Each policy iteration comes with its potential outcome, shifting the geospatial distribution of area risk posed by these cull populations.

The redistribution of interactions generates new indirect connections between state and processing facility populations, potentially allowing disease to spread in previously unimaginable ways. Suppose disease introduction triggers a regionalized standstill of movements, ensuring all infected animals are within the defined closure area and that no infected animals exist within the marketing populations is of extreme importance. Suppose unknown infected animal movement is allowed to happen and interacts with one of the many independent cull sow herds. In that case, this work suggests that the stop movement order is likely to increase the probability of an indirect connection between that infected population and numerous states, as previous relationships now change, allowing a disease to move seemingly randomly throughout the industry.

## Figures and Tables

**Table 1:** Results of the fitted PPML models for all three scenarios. Results reported as regression coefficient (Standard Error), *p* < 0.001 ***.

Variable	Normal Function	North Carolina Closure	Missouri Closure
Distance	−1.158 (0.091) ***	−1.302 (0.088) ***	−0.783 (0.120) ***
Facility’s Weekly Slaughter Capacity	1.053 (0.080) ***	1.124 (0.084) ***	0.4299 (0.125) ***
State’s Weekly Slaughter Capacity	0.803 (0.081) ***	0.550 (0.081) ***	-
Regional Basis	-	-	-

**Table 2:** Results of model validation for F1 closure. Results reported as regression coefficient (Standard Error), *p* < 0.001 ***.

Variable	F1 Closure Scenario	F1 Closure Actual
Distance	−0.744 (0.108) ***	−0.899 (0.214) ***
Facility’s Weekly Slaughter Capacity	0.950 (0.092) ***	1.00 (0.186) ***
State’s Weekly Slaughter Capacity	0.334 (0.089) ***	-
Regional Basis	-	-

## Data Availability

Data is not available for reporting due to confidentiality.
